# *Giardia duodenalis *in feedlot cattle from the central and western United States

**DOI:** 10.1186/1746-6148-5-37

**Published:** 2009-10-02

**Authors:** Bruce R Hoar, Robert R Paul, Jennifer Siembieda, Maria das Gracas C Pereira, Edward R Atwill

**Affiliations:** 1 Department of Medicine and Epidemiology, School of Veterinary Medicine, University of California-Davis, Davis, CA 95616, USA; 2 Department of Population Health and Reproduction, School of Veterinary Medicine, University of California-Davis, Davis, CA 95616, USA; 3 810 Schreider Street, Fort Detrick, Maryland 21702-5000, USA

## Abstract

**Background:**

*Giardia duodenalis *is a ubiquitous protozoan parasite that has emerged as a significant opportunistic human pathogen. *G. duodenalis *may have a deleterious effect on animal growth and performance, therefore its potential as a production limiting organism should not be discounted. We therefore undertook this study to determine management and environmental factors in feedlots that influence the prevalence and environmental load of *G. duodenalis *cysts in fecal material deposited by feedlot cattle in the central and western United States.

**Results:**

Twenty two feedlots from 7 states were included in the study, and up to 240 fecal samples were collected from pen floors of up to 6 pens per feedlot. *Giardia duodenalis *cysts were identified and counted using direct immunofluorescent microscopy. The estimated overall point prevalence of *G. duodenalis *was 19.1%, representing feedlots from a wide range of climates and management systems. Pen-level prevalence varied from 0 to 63.3%, with pen-level shedding estimates ranging from 0 to 261,000 cysts/g feces. Higher environmental temperatures, increased animal density, and increased time in the feedlot were associated with a lower prevalence of *G. duodenalis*. Removing manure before placing a new group of cattle in a pen was associated with a decreased prevalence of *G. duodenalis *in fecal pats. Using coccidiostats as a feed additive was associated with a higher prevalence of *Giardia*.

**Conclusion:**

Management practices could be employed that would limit the probability that feedlot cattle shed *G. duodenalis *in their feces and therefore potentially limit contamination of their environment.

## Background

*Giardia duodenalis *is a ubiquitous protozoan parasite that has emerged as a significant opportunistic human pathogen. At least seven distinct assemblages (A - G) of *G. duodenalis *have been identified [1] however only assemblages A and B are associated with human infections [2]. *Giardia *infections are usually self-limiting in people with normal immune systems, but can be severe in immuno-compromised individuals [3-5]. Since the infectious dose for susceptible humans may be as low as 10 *G. duodenalis *cysts [6] it is important to minimize contact between fecal material containing infectious cysts and water supplies used for municipal, recreational, or irrigation purposes.

While cattle have been found to be primarily infected with the livestock-specific assemblage E genotype, a portion of *G. duodenalis *infections in ruminants have been attributed to either assemblage A mono-infection [7], to mixed infections with both assemblage A and E [8,9], or to infection with assemblage B [10]. This raises uncertainty over the relative importance of cattle manure as a source of transmission of the parasite to humans; however genotyping data suggest that the public health risk is probably minimal since the livestock genotype predominates in cattle [2,3]. *G. duodenalis *infection in dairy cattle had been studied because the confinement practices of dairies has been linked with increased *G. duodenalis *infection and reinfection rates of calves [11,12]. The focus of research then was turned to beef cattle on pastures and *Giardia *cysts were found in agricultural run-off at an increased concentration during peak calving time [13]. Further studies attempted to determine the prevalence of *Giardia *in cattle coming from pastures into a confinement operation [14]. Young cattle are more likely to shed *G. duodenalis *cysts than adult cattle. In particular, previous studies have found that the prevalence of *G. duodenalis *shedding for beef and dairy calves up to 9 months ranges from 17-55% and for adult beef and dairy cattle ranges from 0-17% [13,15-19]. It is uncertain whether infection of ruminants with *G. duodenalis *is associated with clinical disease [7], but infection may be associated with the occurrence of diarrhea and ill-thrift in calves [8,12] and severe weight loss in lambs [20]. An experimental study using SPF lambs has been shown that *G. duodenalis *can have a deleterious effect on animal growth and performance [21], therefore its potential as a production limiting organism should not be discounted.

Much of the variation in reports of prevalence is likely due to the differences in populations of cattle studied, which include the type of operation (beef versus dairy), age distribution of cattle, and variation in management practices [22]. It has been shown that the rate of environmental loading of *G. duodenalis *per animal unit or mass of fecal material is conditional on the underlying age distribution of the herd [15-17]. Other studies found that the prevalence of *Giardia *infection was not associated with factors such as calf management, confinement, manure accumulation, farm hygiene, or duration of the calving season [14,19,23].

Presently, little is known about the prevalence of *G. duodenalis *in cattle in commercial feedlots. Large numbers of cattle are concentrated in feedlots across the United States, leading to high-volume point sources of fecal waste. In order to develop management plans to reduce the potential for waterborne transmission of *G. duodenalis*, a valid estimate of the prevalence of infection and number of cysts present in the fecal material deposited by feedlot cattle is required. Therefore, we undertook this study to determine management and environmental factors in feedlots that influence the prevalence and environmental load of *G. duodenalis *cysts in fecal material deposited by feedlot cattle in the central and western United States.

## Methods

### Feedlot selection and fecal sampling

One to 4 feedlots from selected states that were deemed by collaborators to be representative of feeding operations in those states were identified for inclusion in the study. Factors considered included location, animal capacity, type of cattle fed, and management system. A total of 22 feedlots in 7 states (California, Washington, Colorado, Oklahoma, Texas, Nebraska, and South Dakota) were eventually enrolled. Feedlots in different states were sampled in sequence so that fecal samples were collected from feedlots with a variety of animal husbandry practices both within and across states and across different climatic conditions and seasons. Approximately 240 fecal samples were collected at each feedlot, from between 2 and 6 pens of cattle. Samples were collected from a randomly selected pen of cattle that had recently arrived to the feedlot, a randomly selected pen of cattle nearing their harvest date, and the remaining pens were randomly selected from across the feedlot. One-hundred-gram fecal samples were collected from freshly-deposited fecal pats, placed on ice packs, and shipped by overnight courier to the Veterinary Medicine Teaching and Research Center, Tulare, CA within 24 hours of collection. A questionnaire related to feedlot and pen management was administered to the feedlot manager at the time of fecal sampling. The following data was recorded: number of animals in the pen, temperature and precipitation during the previous month, animal class (heifer, steer, bull, beef, dairy), water source, pen size, days in feedlot, coccidiostats in feed, days between lots of cattle, pen cleaning/scraping frequency, presence of a cover for the cattle, percent concentrate in feed, primary concentrate used, source of cattle (auction, ranch derived, combination), and water/feed trough cleaning frequency and method.

### Detection and enumeration of *G. duodenalis *cysts

Direct immunofluorescent microscopy (DFA) was used for detecting and enumerating *G. duodenalis *cysts as previously described [24]. Briefly, five grams of each fecal sample were mixed with 40 mL of deionized water and washed through folded 2-ply gauze. Fecal suspensions were centrifuged at 1,000 × g for 10 minutes, supernatants aspirated, and the residual fecal sediments resuspended 1:1 (v/v) in deionized water to a final volume of 3 to 5 ml. Ten μl of fecal suspension (average weight = 11.7 mg) was smeared onto glass slides and dried overnight. Detection of cysts was performed using the direct immunofluorescent assay (Merifluor *Cryptosporidium/Giardia *detection kit, Meridian Diagnostic, Inc., Cincinnati, OH). The entire slide was scanned at ×200 magnification. The total number of *G. duodenalis *cysts was recorded for each smear.

To determine percent recovery for immunofluorescent microscopy, we collected three fecal samples from beef feedlot cattle that tested negative for *G. duodenalis *and added purified wild-type dairy calf *G. duodenalis *cysts to a final concentration of either 100, 1000, or 10,000 oocysts/L. Three replicates per concentration per fecal sample were used for a total of 26 fecal samples. The immunofluorescent microscopy procedure was then performed as described above. Percent recovery was estimated by fitting a negative binomial regression model to the observed number of oocysts, with the number of spiked or expected oocysts functioning as the offset variable and fecal identification as a cluster variable due to using three replicates per concentration per fecal sample [25]. The estimated percent recovery for enumerating *G. duodenalis *cysts using the immunofluorescent microscopy procedure was 36.2% (95% CI, 28.4% - 46.2%). The estimated concentration of *G. duodenalis *was therefore calculated by dividing the number of cysts observed by the average amount of fecal material on each slide (0.0117 grams) multiplied by 0.362, the estimated cyst recovery rate.

### Statistical Procedures and Modeling

The prevalence of *G. duodenalis *was calculated by type of feedlot pen, management practices, and climate factors. Pen order was categorized into three groups; entry pens (nutrition based primarily on forages), middle pens (feedlot diets transition from high roughage to high concentrates), and exit pens (diets containing a high proportion of concentrates). The concentration of cysts in each positive sample was placed into categories of fecal cyst load. The prevalence of *G. duodenalis *positive samples was calculated for each category of fecal cyst load by pen order. Differences in proportion *G. duodenalis *positive between groups were assessed using a univariate logistic regression.

A logistic regression model was created using a commercial software program (Egret^® ^for Windows, Cytel Software Corporation, Cambridge, MA) to identify cattle characteristics (such as sex, breed, and source), farm management practices, and climate factors associated with the presence of *G. duodenalis *cysts in fecal samples from feedlot cattle. Descriptive statistics were performed to determine variable ranges, the extent of outliers, and to check for normality of continuous variables. All potential risk factors were initially screened for their association with the presence of *G. duodenalis *cysts using univariate logistic regression. Variables with a Pearson's chi-square statistic of p ≤ 0.25 were offered entry into the model building process. Two-way interaction variables were created from this list and offered for entry. A forward stepping approach was used to develop the logistic regression model, with p ≤ 0.05 as determined by a likelihood ratio test set as the criterion for inclusion of the variable in the final model. The variable "pen" was included as a random effect term in the final model because fecal samples from the same pen were not considered to be independent.

## Results

A total of 5,260 fecal samples were collected from 22 feedlots in California, Colorado, Nebraska, Oklahoma, South Dakota, Texas, and Washington. The overall proportion of fecal samples containing detectable *G. duodenalis *cysts was 19.1% (1,006/5,260); prevalence within pens varied from 0% to 63.3%, while prevalence within feedlots varied from 5.4% to 43.6% (Table 1). The proportion of *G. duodenalis *positive fecal pats in entry pens was 26.1% (n = 1401), in mid-duration pens was 17.2% (n = 2488), and in pens nearing harvest was 15.5% (n = 1371). The prevalence for entry pens was significantly higher than in middle and exit pens (p < 0.001).

**Table 1:** Prevalence and intensity of *Giardia duodenalis *cysts in fecal samples from beef cattle located on 22 feedlots from central and western United States.

**State/Month of sampling**	**Feedlot prevalence^1 ^(sample size)**	**Range of prevalence by pen^2^**	**Range of intensity of fecal shedding by pen^2 ^(cysts/g feces)**
**Texas**			
January	9.3 (226)	0.0 -- 33.3	0 -- 31,960
April	5.4 (242)	0.0 -- 8.3	0 -- 3,513
September	11.0 (236)	0.0 -- 16.7	0 -- 15,429

**Oklahoma**			
January	23.8 (240)	1.7 -- 56.7	46 -- 26,955
May	21.7 (240)	11.7 -- 36.7	436 -- 51,430
January	14.6 (239)	0.0 -- 31.7	0 -- 2,089

**California**			
August	16.7 (245)	13.3 -- 20.0	4,519 -- 58,663
February	20.0 (240)	10.0 -- 38.3	298 -- 5,166
September	7.5 (240)	3.3 -- 11.7	390 -- 3,605
January	6.4 (235)	3.4 - 13.3	47 -- 16,141

**Washington**			
October	34.2 (240)	25.0 -- 52.5	1,722 -- 261,710

**Colorado**			
September	20.4 (240)	11.7 -- 33.3	1,079 -- 219,406
March	30.9 (236)	20.0 -- 45.0	1,446 -- 7,783
July	13.9 (244)	8.3 -- 16.9	184 -- 22,298
January	18.8 (240)	5.0 -- 45.0	115 -- 22,248

**Nebraska**			
November	25.0 (240)	3.3 -- 63.3	161 -- 144,327
April	19.5 (236)	8.3 -- 30.0	941 -- 7,278
September	12.2 (238)	0.0 -- 25.0	0 -- 39,698

**South Dakota**			
August	23.3 (240)	11.7 -- 38.3	918 -- 10,952
March	43.6 (243)	26.7 -- 55.0	3,306 -- 7,026
July	25.8 (240)	21.7 -- 28.3	689 -- 2,112
January	15.8 (240)	10.0 -- 16.7	276 -- 1,515

**Overall total**	**19.1 (5,260)**		

^1 ^Fecal samples from each feedlot were collected from two to six pens of cattle, stratified by time on feed (entry, middle, exit)

^2 ^Prevalence of fecal samples with *Giardia duodenalis *cysts and their mean intensity (cysts/g feces) calculated at the level of pen

**Figure 1:**
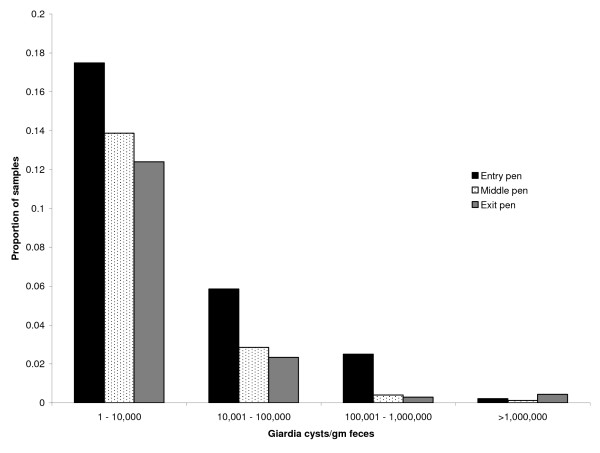
**Proportion of *Giardia duodenalis *positive fecal samples from central and western United States, classified by pen order and concentration of cysts**. Entry pens are where cattle first enter the feedlot from pasture, middle pens are where feedlot diets transition from high roughage to high concentrates, and exit pens are where cattle are fed high concentrate rations immediately prior to harvest. Negative fecal samples are not shown.

The distribution of fecal concentration of *G. duodenalis *by pen type is shown (Figure 1). There were a greater proportion of fecal samples from entry pens in each fecal concentration category, except for the greater than 10^6 ^category. Twelve fecal samples (0.2% of all samples) were estimated to have more than 10^6 ^*G. duodenalis *cysts per gram of feces. These fecal samples were from 6 different feedlots; four of these high intensity fecal samples were from the same pen of exit cattle.

**Figure 2:**
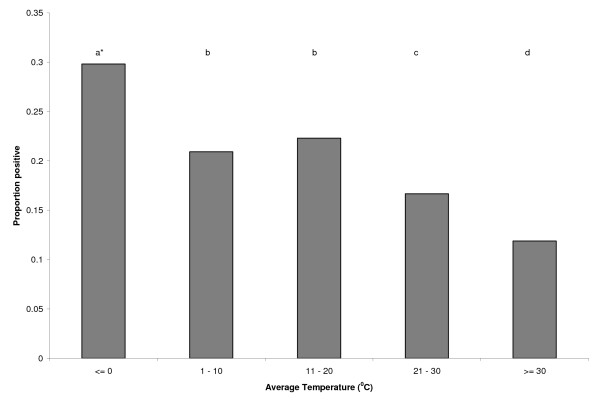
**Proportion of fecal samples from feedlot pen floors positive for Giardia duodenalis by average temperature in the prior month from central and western United States**. Categories with different superscripts are significantly different (P < 0.05).

The proportion of fecal samples positive for *G. duodenalis *was inversely associated with temperature; prevalence was greatest in feedlots which had had the coldest temperatures in the past month, and least in feedlots which had had the hottest temperatures in the past month (Figure 2).

Almost all feedlots had a dirt surface, used feed bunks for feeding (as opposed to feeding on the dirt/floor), fed corn as their primary concentrate, had no cover for the cattle, and used wells as their primary water source; these variables are not included in the logistic regression model due to a lack of variation. The final logistic regression model included number of days in the feedlot, average temperature during the previous month, animal density within pens, whether manure was removed between lots of cattle, and whether a coccidiostat was included in the ration (Table 2). Several factors were not associated with a sample testing positive for *G. duodenalis *including type of roughage fed, percent energy concentrate in current ration, feed bunk cleaning methods, source of cattle, and whether cattle come from single or multiple sources.

**Figure 3:**
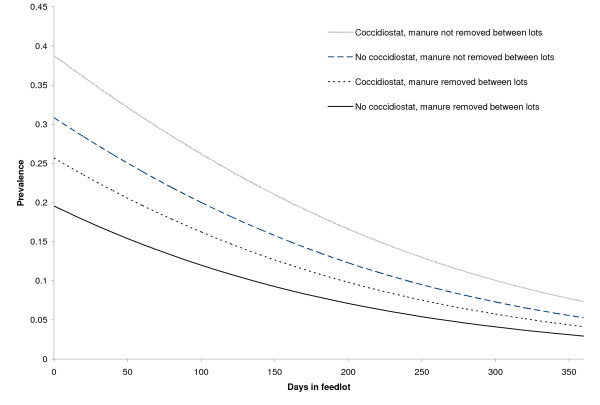
**Predicted *G. duodenalis *prevalence in feedlot cattle from central and western United States by number of days cattle have been in the feedlot**.

**Figure 4:**
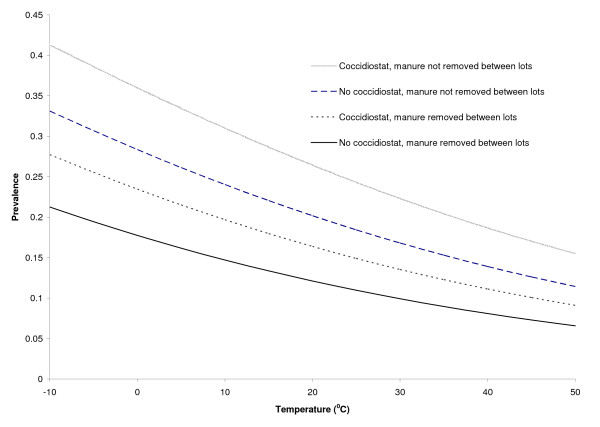
**Predicted *Giardia *prevalence in feedlot cattle from central and western United States by average temperature of the previous 30 days**.

The predicted prevalence of *G. duodenalis *by the number of days in the feedlot and by temperature is shown in Figures 3 and 4 (based on the final regression model). These show that increasing days in the feedlot and increasing ambient air temperature were associated with a reduction in *Giardia *prevalence, that feeding a coccidiostat increases the prevalence over not feeding a coccidiostat, and that manure removal prior to placing cattle in a pen is associated with reduced prevalence of infection.

## Discussion

In this sample of fecal material from feedlots in the central and western United States, the point prevalence of *G. duodenalis *was 19.1%, with 26.1% of samples from newly-arrived cattle and 15.5% of samples from cattle near harvest testing positive. While the cumulative infection rate with *Giardia *may reach 100% in range beef calves [14], previous studies of beef cattle on pasture reported point prevalence of *G. duodenalis *in 6.5% and 11% of fecal samples [23,26]. This suggests that feedlot confinement may create the conditions that allow for a higher point prevalence of *Giardia *infection as compared to pasture settings, especially when cattle first enter the feedlot. As cattle remain in the feedlot, prevalence is reduced. A longitudinal study in calves found a cumulative prevalence of 100%, with 85% prevalence in the first month, decreasing to 21% prevalence in months 6-7 [14]. While an individual animal seems very likely to become infected at some point in their life, the current study provides a valuable estimate of point prevalence at various times during the feeding period.

Results from this study indicate that hot climates are associated with reduced prevalence of *G. duodenalis *in pen fecal deposits; our model predicted that as temperature increased the odds of detecting *Giardia *cysts decreased by 2.2% per °C. This may result from the desiccation of *Giardia *cysts in the fecal material by the high temperatures, thereby reducing transmission among cattle within a pen and the probability that a freshly collected fecal pat would contain cysts. Bingham et al. studied the physical factors involved with excystation of *Giardia *and viability in varying temperatures [27]. Cysts survive up to 77 days in 8°C, 5-24 days in 21°C, and not longer than 4 days in 37°C under laboratory conditions. The internal temperature of fecal pats can increase to as high as 70°C [28] which further affects the viability of *Giardia *cysts and decreases the potential for infection of penmates. Other researchers have demonstrated that *Giardia *cyst viability was compromised by shearing forces generated during freeze-thaw cycles, while lower temperatures were protective of cysts [29]. The prevalence of *Giardia *in feedlots that had an average temperature range of 1 to 10°C, which may reflect potential freeze/thaw conditions, was significantly lower than feedlots with an average temperature range below freezing (Figure 2). If fewer cysts are viable infection rates in a feedlot are likely to be reduced. Another possible explanation is that at the coldest temperatures, animals are more likely to cluster together more closely, thereby increasing the effective animal density and transmission potential. The regression model supports the studies above by demonstrating a negative relationship between temperature and *Giardia *positive cattle (Figure 4). All of the feedlots included in this study were outdoors (typical of central and western United States), therefore we cannot comment on the possible effect of temperature in indoor facilities.

Increased pen area per animal was found to have a slight positive association with prevalence of *G. duodenalis *(Table 2). While this finding is counterintuitive, the magnitude of the association was small, and may represent the effect of other unmeasured factors that confound the relationship between animal density and shedding.

**Table 2:** Results of a random effects logistic regression model examining factors associated with presence of *Giardia duodenalis *in cattle feces from feedlots from central and western United States.

**Predictor**	**Odds Ratio (95% confidence interval)**	***P *- value**
**Days in feedlot**	0.994 (0.991, 0.997)	<0.001
**Avg. Temperature (°C)**	0.978 (0.965, 0.991)	<0.001
**Pen density (m^2^/head)**	1.001 (1.000, 1.002)	0.02
**Coccidiostat in feed**		
Yes	Referent category	
No	0.704 (0.501, 0.989)	0.04
**Manure removal between lots**	Referent category	
Yes	1.83 (1.31, 2.57)	
No		<0.001

Coccidiostats, which include monensin, lasalocid, and decoquinate, are feed additives commonly incorporated into feedlot rations in order to control infection with various *Eimeria *spp. However, the primary reason for including most coccidiostats in feedlot rations is to improve feed to gain conversion. In this study, feeding a coccidiostat in the ration was associated with an increased prevalence of infection with *G. duodenalis*. These feed additives selectively inhibit certain ruminal microorganisms, thereby altering fermentation efficiency and end products available for absorption and performance [30]. We speculate that the alteration in the rumen environment may influence survival and reproduction of this parasite. Another possible explanation would be that if the feedlot believes that use of coccidiostats is warranted, then conditions may be appropriate for transmission of coccidiosis, and these conditions may be similar for transmission of giardiasis. This observation warrants further investigation.

Removing manure prior to placing a new group of cattle in a pen was found to be associated with a decreased prevalence of *G. duodenalis *(Table 2). Physical removal of potentially infective cysts before exposing a new group of cattle to the pen is a management practice that could reduce the potential for cyst transmission.

While there are reports examining the environmental load of *Cryptosporidium parvum *[22,23,25], there are few published studies measuring the environmental load of *Giardia *cysts to compare the results from this research. The current study found that feedlot cattle can shed *G. duodenalis *in the range of tens of millions to hundreds of millions of cysts per animal per day. Ralston et al. performed a longitudinal study that demonstrated a decline in prevalence of *G. duodenalis *over time and attributed the higher prevalence in young cattle entering the feedlot to a temporary relaxation of immunity from the stresses of weaning and being shipped to a feedlot [14]. The current logistic regression model supports this hypothesis by showing a negative correlation between days in the feedlot and the number of *Giardia *positive cattle (Figure 3). Further support is confirmed in Figure 1, demonstrating a larger number of cattle infected with *Giardia *in the entry pens, as compared with middle and exit pens. These findings may indicate that many animals stop shedding *Giardia *cysts as the animal ages and the duration of their stay at a feedlot lengthens.

We found a small percentage of fecal pats containing a high concentration of cysts. These cattle were found from all stages of the feeding period, which may provide evidence for the existence of *Giardia *"super-shedders". Researchers have noted this same phenomenon of cattle shedding high concentrations of *Escherichia coli *O157:H7 and *Mycobacterium avium *spp. *paratuberculosis *and have put forth the concept of super-shedders and heavy-shedders, respectively [31-35]. While the proportion of cattle shedding greater than 1 million cysts/g feces was small, these cattle potentially represent a significant source of cysts that could potentially contaminate the feedlot environment.

## Conclusion

Recent evidence indicates that cattle are most commonly infected with the non-zoonotic livestock genotype of *G. duodenalis *which limits their role as reservoirs of giardiasis in humans [2,3,26]. Nevertheless, given the uncertainty over whether infection with *G duodenalis *has any adverse clinical consequences in cattle, knowledge of prevalence and risk factors for shedding is important in order to generate management plans. While grazing on pasture may not concentrate cattle and cysts, a feedlot setting may create a sufficient concentration of fecal deposits on a limited area of land; therefore it is recommended that feedlot fecal waste management plans be implemented in order to minimize the potential for environmental contamination with this and other potential pathogens.

## Authors' contributions

BH helped conceive the project and assisted in writing initial grant application, provided input and advice on statistical modeling, and revised the manuscript. RP assisted in developing statistical models, drafted the initial manuscript. JS performed initial descriptive statistics, and provided input into the initial manuscript. MP performed all laboratory analyses, provided editing for manuscript. EA wrote final draft of grant, supervised all sampling and data collection, assisted in statistical concepts, and edited the final manuscript. All authors read and approved the final manuscript
